# Clinical, Therapeutic, and Prognostic Experience in Patients With Glioblastoma

**DOI:** 10.7759/cureus.29856

**Published:** 2022-10-03

**Authors:** Michel Mondragon-Soto, Luis A Rodríguez-Hernández, Sergio Moreno Jiménez, Juan Luis Gómez Amador, Axayacatl Gutierrez-Aceves, Humberto Montano-Tello, Ignacio Reyes-Moreno, Jose Santos-Zambrano, Elvira Castro-Martinez, Alberto Gonzalez-Aguilar

**Affiliations:** 1 Neurological Surgery, Instituto Nacional de Neurologia y Neurocirugia, Tlalpan, MEX; 2 Neurosurgery, Instituto Nacional de Neurología y Neurocirugía Manuel Velasco Suárez, Mexico City, MEX; 3 Neurological Surgery, American British Cowdray (ABC) Medical Center, México City, MEX; 4 Neurocirugía, Instituto Nacional de Neurología y Neurocirugía Manuel Velasco Suárez, Mexico City, MEX; 5 Neuroradiosurgery, Neurology and Neurosugery National Institute, Mexico City, MEX; 6 Neurology, Instituto Nacional de Neurologia y Neurocirugia, Ciudad de Mexico, MEX; 7 Neuroradiosurgery, Neurology and Neurosurgery National Institute, Mexico City, MEX; 8 Neurology, Neurology and Neurosurgery National Institute, Mexico City, MEX; 9 Neurology, Gea Gonzalez Hospital, Mexico City, MEX; 10 Neurooncology, Neurology and Neurosurgery National Institute, Mexico City, MEX

**Keywords:** brain tumors (primary or brain metastasis), neurologic complication of cancer or immunotherapy (cart), radiotherapy, therapy, survival, glioblastoma

## Abstract

Background: Glioblastoma (GB) represents the most aggressive type of glioma with a poor prognosis despite the therapies used. As of today, data availability for therapeutic and prognosis experiences is limited. The cornerstone for this study is to create a framework overview of Mexico´s experience throughout 17 years of research.

Methods: Retrospective analysis from 2000 to 2017 including patients with a histological diagnosis of GB was performed. Data were collected from the ABC Medical Center and the Neurology and Neurosurgery National Institute.

Results: One hundred and thirty-seven patients were included with a mean age of 54 years. Histological diagnosis was made in all patients, of which 58.1% had a total resection, 31.6% had a partial resection, and 10.3% of them underwent biopsy. In all cases, patients received treatment under the following conditions: 10 patients were treated exclusively with stereotactic radiotherapy (RT). In 55 patients, a combination of RT and TMZ was used, the other 40 patients received RT plus CBP. Eighteen patients RT added to nitrosourea medication and lastly, 14 patients received a combination of RT/TMZ and Bevacizumab, a monoclonal antibody that inhibits the formation of blood vessels (BVZ). The progression-free survival (PFS) and overall survival (OS) were higher in the RT/TMZ/BVZ group (16.5 to 22.9 months) and the RT/TMZ group (11 to 17 months), the prognostic parameters included: Isocitrate dehydrogenase 1 mutation (*IDH1*), usage of BVZ and TMZ in the PLS and OS, considering as well, age range (<70 years) as a favorable prognostic factor.

Conclusions: GB represents the most frequent intracranial neoplasia. Combined fractionated stereotactic RT added to Temozolomide and Bevacizumab received in our population reports favorable and superior results compared to the ones described in the literature. Further studies are necessary to know the biological behavior of our population.

## Introduction

Central nervous system tumors (CNSTs) are the tenth leading cause of estimated new cancer cases and the 12th leading cause of death in adults worldwide [1]. Estimated 22,850 new cases and 15,320 deaths in 2015 [2]. In the United States, CNSTs contribute to one of the highest incidence rates of cancer [3]. Gliomas are the most common type of CNST presentation in adults; it represents 12% to 15% of intracranial neoplasms and 50% to 60% of astrocytic tumors [4].

Glioblastoma (GB) is responsible for 12,000 annual deaths in the United States and only 9% of patients are alive at a five-year follow-up [4,5]. As of today, once maximum surgical resection of the tumor has been performed, gold standard care is based on the results of the phase three study of Stupp, with concomitant and adjuvant radiotherapy (RT) with Temozolomide (TMZ) [6,7]. Current data for distribution, frequency, age, and sex regarding CNSTs tumors in Mexico is unknown, as are the clinical, imaging, and treatment characteristics and outcomes of the Mexican population.

After 30 years, GB was the first brain tumor sequenced by the Cancer Genome Atlas (TCGA) [8]. A number of mutations and molecular pathways in its pathogenesis had been discovered. Unfortunately, therapeutics are very limited despite multiple studies, survival has been extended to 20.5 months [9]. The purpose of this study is to describe sociodemographic and clinical features, diagnosis, treatment and prognostic factors of GB at a tertiary health care level center in Mexico.

## Materials and methods

The database of two tertiary health care level centers in Mexico (National Institute of Neurology and Neurosurgery; INNN and American British Cowdray Medical Center; ABCMC) was reviewed. Inclusion criteria included: patients with a histopathological diagnosis of GB who were treated at one of the two centers. This manuscript compares recorded data retrospectively throughout 17 years (2000-2017) regarding diagnosis, location of the lesion, type of surgical resection performed, six-month follow-up, and sociodemographic and clinical features. Formal written informed consent was not required with a waiver by the appropriate International Review Board (IRB) and/or national research ethics committee in accordance with the provisions of the Regulations of the General Health Law of Mexico in the field of Health Research in its article 17, the present research study is considered to be of less than minimal risk, since only information was taken from the electronic file, in fact, data that could allow the identification of any of the patients are never disclosed. Present survival analysis considered initial neurosurgery intervention as day number one. Censoring screening was initially based on progression-free survival (PFS), documented death, or last follow-up day. A multivariate Cox study was estimated for prognostic factors. Results were considered statistically significant if p ≤ 0.05.

## Results

A total of 597 cases were reviewed in both centers. Only 137 patients met the inclusion criteria. The age range was 23-84 years with a mean of 53.6 years. By gender, men represented 69.1% and women 30.9%. The mean Score for the Karnofsky scale (KPS) was 83 (range 20-100). The most frequent clinical presentation was a motor deficit, cognitive impairment, and headache. Summary of initial symptoms listed in Table 1.

**Table 1:** Clinical manifestations.

Clinical Manifestation	N	%
Seizures	32	23.5
Status Epilepticus	4	2.9
Headache	50	36.8
Cognitive Impairment	57	41.9
Awareness Deficit	24	17.6
Motor Deficit	66	48.5
Paresthesia	27	19.9
Vision Imparment	36	26.5
Cerebellar deficit	14	10.3
Cranial nerves deficit	5	3.7
Amaurosis	3	2.2

Tumor´s most frequent locations were demonstrated within the frontal and temporal lobes prevalently affected. Total resection was achieved in 58.1%, partial resection in 31.6%, and biopsy in 10.3%. There were 85 patients with tested for Isocitrate Dehydrogenase type 1 mutation by immunohistochemistry (IDH1) where a total of 19 patients tested positive for it, meanwhile, 66 patients had a negative immunological expression.

**Figure 1:**
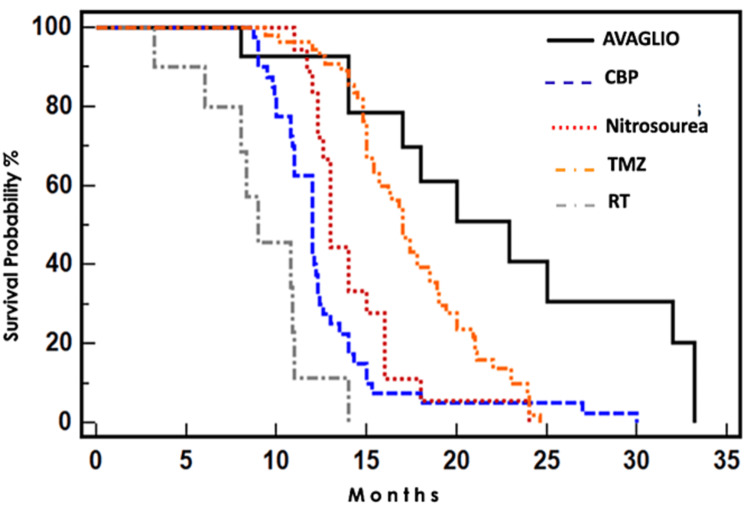
OS per treatment sample group. OS: Overall Survival

**Figure 2:**
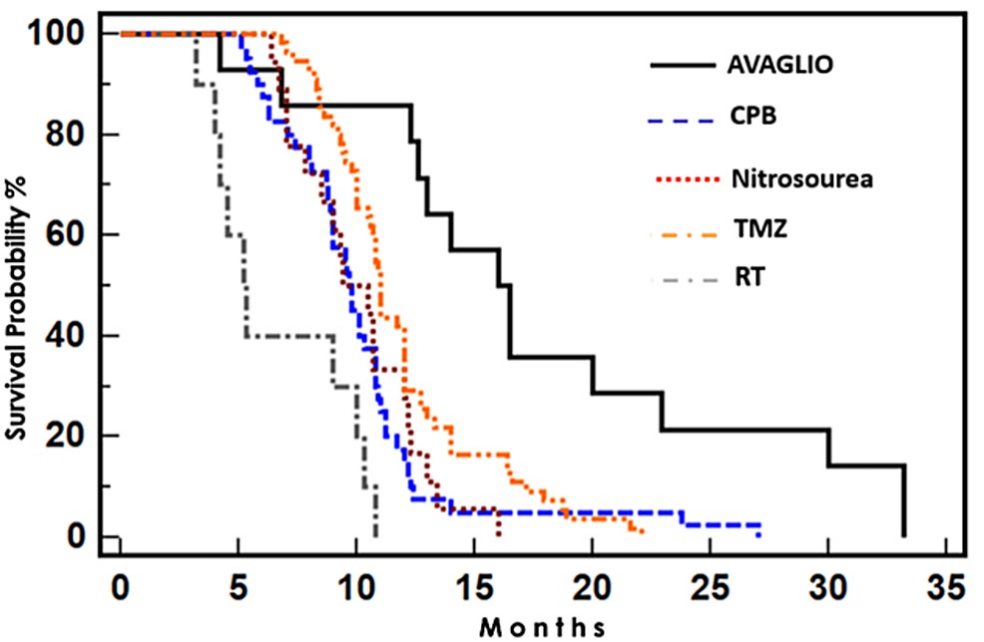
PFS per treatment sample group. PFS: Progression-free survival

Regarding treatment, all patients received RT. Protocol included: 60 Grays (Gys) for 30 sessions administering 2 Gys per session. The two most frequently pharmacological therapeutic strategies prescribed with RT were TMZ for 55 patients and CBP for 40 patients based on immunohistochemistry. The rest of the samples were administered as described in the AVAglio protocol with the addition of Lomustine or Carmustine (Nitrosoureas Group) and triple therapy with Radiotherapy, TMZ, and Bevacizumab (BVZ) [10,11]. In this light, PFS was superior with the triple combination of RT, TMZ, and BVZ (16.5 months), followed by RT with TMZ (11 months) (Figures 1, 2)*.*

Overall survival (OS) was superior following the AVAGLIO treatment regimen (22.9 months), followed by the TMZ group (17 months) (Table 2)*.*

**Table 2:** Variability comparison within patients. RT: Radiotherapy; TMZ: Temozolomide; CBP; Carboplatin; BVZ: Bevacizumab; OS: Overall Survival; PFS: Progression-Free Survival; KPS: Karnosfsky Scale; IDH: Dehydrogenase type 1 mutation by immunohistochemistry; GTR: Glioblastoma total resection.

Treatment	Number of patients	PFS (months)	OS (months)	Age	GTR (%)	KPS	IDH
RT Only	10	5.2	9	55	6 (60)	82	1
RT + TMZ	55	11	17	54	29 (52.7)	83	9
RT+ CBP	40	9.4	12	53.9	24 (60)	80	3
RT+ Nitrosourea	18	9.7	13	54.7	11 (61)	86	1
RT+TMZ+BVZ	14	16.5	22.9	50.8	10 (66.6)	81	5

**Figure 3:**
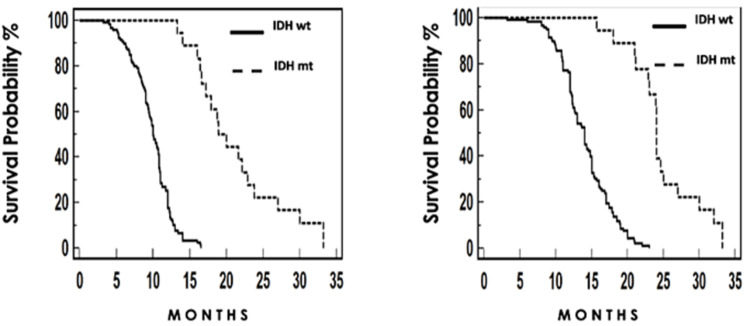
Clinical experience based on IDH 1 mutation. Left: Progression. Right: Overall survival.

PFS and OS were analyzed, finding statistical differences in both groups of patients carrying the mutation: mutated IDH (TLE 18.9 months and SG 24 months) vs IDH wild type (TLE 12 months and SG 14 months) (Figure 3)*.*

The distribution of favorable prognostic factors (KPS, GTR, Age, and IDH) between the treatment groups were analyzed without finding significant differences, represented in Table 3.

**Table 3:** PFS and OS analysis. TMZ: Temozolomide; BVZ: Bevacizumab; OS: Overall Survival; PFS: Progression-Free Survival; KPS: Karnosfsky Scale; IDH: Dehydrogenase type 1 mutation by immunohistochemistry; GTR: Glioblastoma total resection.

Analysis		
Overall Survival		
Variable	p	IC 95%
KPS	0.5386	0.5612 to 1.3509
GTR	0.1798	0.8915 to 1.8610
>70 years	0.0193	0.2239 to 0.8729
IDH1	0.0021	0.0255 to 0.1672
TMZ	0.0069	0.1606 to 0.3750
BVZ	0.0045	0.0491 to 0.2638
Progression Free Survival		
Variable	p	IC 95%
KPS	0.5196	0.7596 to 1.7268
GTR	0.7568	0.7468 to 1.4950
>70 years	0.1687	0.8213 to 3.1326
IDH1	<0.0001	0.0102 to 0.1140
TMZ	0.0151	0.4351 to 0.9127
BVZ	<0.0001	0.0926 to 0.3984

We performed Cox multivariate analysis for known prognostic factors, as well as the treatment usage comparison for TMZ and BVZ concluding the presence of the IDH1 mutation plus the usage of an antiangiogenic agent as a good prognostic factor per se and age as a favorable prognostic factor (<70 years) itself. The rest of the variables were not significant (KPS and GTR).

## Discussion

GB is the most common primary neoplasm of the CNS, despite the decades of research, the positive outcome is still limited. Current advanced treatments for its management are TMZ (First Line), BVZ (Second line), and intratumoral Carmustine (Gliadel) gathering together a maximum reported in phase 3 studies survival of 20.5 months [12].

Surgical GB treatment has evolved in recent years. The main purpose of tumor removal is diagnostic confirmation and mass effect reduction, which is complicated by its infiltrating nature and rapid growth [12,13]. Other treatment modalities such as RT and chemotherapy (CT), to prolong survival time had been implemented.

In 2005, Stupp et al. published the results of a randomized phase III study that compared surgery followed by RT against surgery followed by RT and concurrent TMZ. In this light at a six-month follow-up, results concluded an increase in progression-free survival, median survival, and OS for the second group sample that received CT. The primary sample reported median survival for 12 months and OS throughout two years of 10.4%. Meanwhile, the secondary group administered with CT reported median survival of 15 months and OS of 26.5% respectively. All differences documented as statistically significant (p<0.001) created the framework for a new standard treatment. More recently, with a five-year follow-up, the benefit of the sample that added temozolomide is maintained, with a five-year survival of 9.8% versus 1.9% for exclusive RT [7-14].

Patients documented for this manuscript are considered pre-TMZ. In Mexico and Latin America there are case reports, a series of small cases about the therapeutic experience in GB, however, our health care system experience has limited accessibility for treatment and even more for the development of appropriate treatment protocols.

Patients are treated with resources available depending on their primary healthcare provider, revealing great heterogeneity and making it difficult to gather these experiences together for analysis with greater methodological power. Clinical experience for sociodemographic data (age and sex) correspond to what is reported in the literature, with the most frequent age of presentation in the 6th decade of life and a predominance in men [11-15]. The clinical symptoms of presentation are motor deficit, cognitive impairment, and intracranial hemorrhage (ICH). Empero, histopathological diagnosis was reached in 58.1% of the cases, which represents a higher percentage than reported in the literature where complete excision is performed in approximately 40% [7-15]. The reason for this percentage to be higher within our sample can be explained by a selection bias in both institutions, particularly at the INNN, since it represents a third-level center that serves the entire country and because of the type of care that it provides. Primarily focus on the population without health insurance with strict screening for study, diagnosis, and follow-up. Regarding the lesion location, the frontal and temporal lobe predominated, being the distribution in terms of the hemisphere, right over the left side. PFS and OS analyses revealed a benefit with the addition of chemotherapy.

The PFS and OS within this review had a higher survival rate compared with the reported in the literature with RT and CT together, particularly when TMZ (PFS 11 months, OS 17 months) or TMZ + BVZ (PFS 16 months and OS 22.9 months) were used. It can be explained by a selection bias, ABCMC is a private health care center, so that, patients can afford facilitates to choose complementary techniques to surgery as well as the type of adjuvant treatment that is required, in the case of the INNN it attends to the entire population of the country without health insurance. Considering this, the selection criteria for patients at the INNN are very rigorous.

We consider that the good results presented in our series are directly impacted by the tight screening and inclusion criteria within both centers. Molecular population genetics could play a role in the response to treatment and prognosis, nevertheless, genomic studies are necessary to sequence our population and be able to confirm this hypothesis. IDH1 was not measured in all patients, but the group of carrier patients showed a PFS of 18.9 months and OS of 24 months, which corresponds to what has been published in the literature [16].

Favorable prognostic factors in GB are extensive excision [17,18], age [6-19], KPS [20], IDH [21,22], and methylation of the methyl guanine methyl transferase (MGMT) promoter [23,24]; meanwhile, in the present study, the favorable prognostic factors were the use of CT with TMZ or TMZ/BVZ, the IDH1 status in comparison to PFS and OS. Age was a favorable prognostic factor (<70 years), Empero, the rest of the factors were not statistically significant which can be explained by the retrospective nature of this study in addition to the selection bias of the institutions, unfortunately, MGMT methylation is a complicated, expensive technique that makes it inaccessible within our media.

The present work is one of the few published works about the clinical, therapeutic and prognostic experience of Mexican patients with GB, the treatment is effective in our population and is superior to the reported in the literature. The phenomenon is explained by a strict high selection of patients that, instead of considering selection error, encourage Mexico´s and Latin America´s public and private health care system to improve the selection guidelines of patients, since good results and longer survival times were demonstrated. More studies are necessary to know the frequency of this condition, as well as the prognostic factors and standardize therapeutics guidelines [25].

## Conclusions

The GB represents the most frequent and aggressive intracranial neoplasia. Combined fractionated stereotactic RT added to TMZ and Bevacizumab received in our population reports favorable and superior results compared to the ones described in the literature. Without a doubt, further studies on Mexican patients are necessary to know the biological behavior of this population.
